# Isolation and Characterization of Bacteria with High Electroactive Potential from Poultry Wastewater

**DOI:** 10.3390/biology12040623

**Published:** 2023-04-20

**Authors:** Aliya Temirbekova, Zhanar Tekebayeva, Aslan Temirkhanov, Dinara Yevneyeva, Azamat Sadykov, Kulyash Meiramkulova, Timoth Mkilima, Akhan Abzhalelov

**Affiliations:** 1 Laboratory of Microbiology, Republican Collection of Microorganisms, Ministry of Healthcare of the Republic of Kazakhstan, 13/1 Valikhanov Str, Astana 010000, Kazakhstan; 2 Department of General Biology and Genomics, L.N. Gumilyov Eurasian National University, Satpayev Str. 2, Astana 010000, Kazakhstan; 3 LLP Astana Bioscience Business Centre, Sh. Kosshygululy Str. 21, Astana 010000, Kazakhstan; 4 Department of Environmental Engineering and Management, L.N. Gumilyov Eurasian National University, Satpayev Str. 2, Astana 010000, Kazakhstan; 5 Department of Civil Engineering, L.N. Gumilyov Eurasian National University, Satpayev Str. 2, Astana 010000, Kazakhstan; 6 Department of Management and Engineering in the Field of Environmental Protection, L.N. Gumilyov Eurasian National University, Satpayev Str. 2, Astana 010000, Kazakhstan

**Keywords:** extracellular electron transfer, gut microbiome, electroactive microorganisms microbial fuel cell, voltage, electricity, anode-respiring bacteria, chemical oxygen demand

## Abstract

**Simple Summary:**

In order to counter the increased deposition of greenhouse gases in the atmosphere, which has resulted in significant climatic changes, the production of alternative fuels to replace conventional fossil fuels has become necessary due to the rapidly diminishing concentration of fossil fuels and the rising global demand for energy. This investigation focused on two different samples that could be used as anolytes to produce energy in single- and double-chamber microbial fuel cells with a graphite electrode. In microbial fuel cells’ energy production, the microbes consume organic substrates, use them in their metabolic processes, and produce valuable products that can be used as fuel to produce energy. The highest voltage outputs from the investigated bacterial strains were generated by strains A1 and A2, at 402 mV and 350 mV, respectively. Strain A6 of the ten different bacterial strains produced the least electricity, with a measurement of 35.03 mV.

**Abstract:**

Natural resources are in short supply, and the ecosystem is being damaged as a result of the overuse of fossil fuels. The creation of novel technology is greatly desired for investigating renewable and sustainable energy sources. Microorganisms have received a lot of interest recently for their potential to transform organic waste into sustainable energy and high-value goods. New exoelectrogens that can transmit electrons to electrodes and remove specific wastewater contaminants are expected to be studied. In this study, we examined three distinct samples (as determined by chemical oxygen demand and pH) that can be used as anolytes to generate power in single-chamber and double-chamber microbial fuel cells using graphite electrodes. Wastewater from poultry farms was studied as an exoelectrogenic anolyte for microbial fuel cell power generation. The study examined 10 different bacterial strains, numbered A1 through A10. Due to their highly anticipated capacity to metabolize organic/inorganic chemicals, the diverse range of microorganisms found in poultry wastewater inspired us to investigate the viability of generating electricity using microbial fuel cells. From the investigated bacterial strains, the highest voltage outputs were produced by strains A1 (*Lysinibacillus sphaericus)* and A2 (*Bacillus cereus*), respectively, at 402 mV and 350 mV. Among the 10 different bacterial strains, strain A6 generated the least amount of electricity, measuring 35.03 mV. Furthermore, a maximum power density of 16.16 1.02 mW/m^2^ was achieved by the microbial fuel cell using strain A1, significantly outperforming the microbial fuel cell using a sterile medium. The strain A2 showed significant current and power densities of 35 1.12 mA/m^2^ and 12.25 1.05 mW/m^2^, respectively. Moreover, in the two representative strains, chemical oxygen demand removal and Coulombic efficiency were noted. Samples from the effluent anode chamber were taken in order to gauge the effectiveness of chemical oxygen demand removal. Wastewater had an initial chemical oxygen demand content of 350 mg/L on average. Strains A1 and A2 decomposed 94.28% and 91.71%, respectively, of the organic substrate, according to the chemical oxygen demand removal efficiency values after 72 h. Strains A1 and A2 had electron donor oxidation efficiencies for 72 h of 54.1% and 60.67%, respectively. The Coulombic efficiency increased as the chemical oxygen demand decreased, indicating greater microbial electroactivity. With representative strains A1 and A2, Coulombic efficiencies of 10% and 3.5%, respectively, were obtained in the microbial fuel cell. The findings of this study greatly advance the field as a viable source of power technology for alternative energy in the future, which is important given the depletion of natural resources.

## 1. Introduction

The need for electricity has risen during the last few years. Fossil fuels and nuclear power are two nonrenewable energy sources which are widely employed worldwide. Fossil fuels are the source of energy that cause the most environmental harm since they continuously release carbon dioxide into the atmosphere, which becomes hazardous when there is an excessive amount of it [1]. Through air pollution and global warming, the quick depletion of fossil fuels has significantly impacted human life [2]. However, several countries have made remarkable efforts to discover a workable solution to the energy problem by concentrating on renewable energy sources, such as solar energy, water energy, and wind energy [3,4,5]. Through the use of highly valuable metal catalysts, these experiments have revealed a novel method for producing energy using a fuel cell [6]. In actuality, there are numerous advantages to adopting fuel cells over other energy providers, including improved efficiency, the absence of mobile components that cause less sonic “pollution”, and the release of zero environmentally harmful gases, such as CO_2_, CO, NO_x_, and SO_x_. On the other hand, these new energy sources have two drawbacks: high cost and low mass production. This is where the concept of a microbial fuel cell (MFC) becomes significantly useful [7].

It is important to remember that the microbial fuel cells (MFCs) approach is a novel bioelectrochemical method that tries to generate energy by utilizing the electrons obtained from biological reactions facilitated by bacteria [8]. It is also worth noting that electroactive microorganisms have sparked a lot of interest in the creation of novel biotechnological systems with minimal environmental impact. They can be applied to the creation of value-added goods, the bioremediation of ecosystems contaminated with metals, and sustainable energy production [9]. It is also important to highlight that due to their extensive freshwater use for the continuous operations of cutting up, washing, and packaging meat, poultry slaughterhouses release enormous amounts of wastewater into the environment. Additional processes used in poultry slaughterhouses, such as scalding, de-feathering, evisceration, and bird washing, also consume a lot of water and produce a lot of wastewater. According to the literature, a 2.3 kg bird will typically drink 26.5 L of water each day [10,11]. The biochemical oxygen demand (BOD) and chemical oxygen demand (COD) measurements show that the effluent from poultry slaughterhouses is significantly polluted with organic materials. Blood, fats, oils, grease, and proteins are among the other components that have significant nitrogen and phosphorus content in poultry slaughterhouse wastewater [12]. Hence, there is a significant risk of the pollution of freshwater sources when inadequately treated poultry wastewater is discharged. The deoxygenation of rivers, groundwater contamination, eutrophication, and the development of water-borne diseases are just a few of the significant environmental and health problems this can lead to [13]. There is a high likelihood of finding novel electrogenic bacteria since poultry wastewater has a high pollutant loading level. Electrogenic bacteria are a class of microorganisms that can transfer electrons extracellularly through the cell envelope to or from electron acceptors such as electrodes, oxide minerals, and other bacteria under anaerobic or microaerobic conditions [14].

The early substrates utilized in the lab were mostly glucose, acetate, or other straightforward substrates to ascertain the behavior of electrode materials, membranes, and other such things, as well as the reactor architecture or microbial activity [15,16,17]. Just recently, investigations employing actual wastewater as a substrate have been carried out. The energy savings from sludge treatment and wastewater aeration were the biggest benefit. The output of sludge by MFCs is also lower than that of anaerobic digesters and aerobic-activated sludge (AS) treatment systems. These have reduced temperature sensitivity, limited electrical installations at sludge treatment facilities, and no energy used for aeration [18]. Fundamentally, wastewater is the most widely used substrate for MFC operations because of its large proportion of organic load and lack of cost. In particular, agro-food wastewater is ideal due to its high biodegradability [19,20,21]. The numerous electroactive and complementary non-electroactive microorganisms convert the chemical energy stored in the chemical components of wastewater or biomass into electrical energy [22]. With its low thermal efficiency, the Carnot thermodynamic cycle in an ideal thermal machine is avoided by this direct conversion of chemical energy to electrical energy. Theoretically, MFCs are comparable to traditional fuel cells in that they can achieve higher efficiency. Additionally, because wastewater is used, it is a renewable energy source, and its “fuel” supply can be controlled relatively more easily than that of wind turbines, where the wind cannot be controlled at all, and photovoltaics, where the sun’s rays cannot be controlled but are predictable [23].

A swine wastewater treatment facility was suggested in the study by Ding et al. [24], based on single-chamber air-cathode MFCs with a solution capacity of 340 mL on a laboratory scale, as well as a separate low-cost flocculation process. According to the findings, an energy recovery rate of roughly 0.664 kWh/m^3^ wastewater mixture was attained. Additionally, 96.6% removal efficiency of COD, 60% removal of ammonia, 37.5 W/m^3^ power density, and 21.6% Coulombic efficiency were attained.

However, despite its potential, the best configuration for MFCs is still being researched, and efforts are now being made to improve its performance by developing more selective proton exchange membranes and alternative electrode materials. Small cells connected in series appear to offer higher potentials than larger reactor volumes [25]. The expense of materials and residential wastewater’s poor buffering capability are currently the main obstacles to the full-scale application of MFCs. Due to this, MFCs have not yet found use in the industry [25]. It is also important to emphasize that the composition of the wastewater and the type of electrode materials utilized can have a significant impact on the effectiveness of these systems. In the MFC, wastewater-growing microorganisms consume the organic substrate and release electrons that are then used to enhance the process of generating energy [26]. In order to break down the substrate and produce energy, the type of microbial population in the biofilm is essential. Several sources of microbial inocula, such as bagasse-based paper mill wastewater [27], fresh sediment [28], dye processing wastewater [29], marine sediment [30] as well as sludge [31] have been used to successfully generate microbial colonies that can transfer their electrons. The choice and appropriateness testing of the inoculum source utilized in the MFC is critical because the kind of microbial population in the biofilm is crucial to both substrate breakdown and energy production. Despite being one of the largest producers of highly polluted wastewater, the poultry industry has unfortunately not been thoroughly examined in relation to MFC energy production. Excreta, feathers, spilled feeds and water, dead birds, cracked eggs, wastewater, litter or bedding materials, and waste from the slaughterhouse and hatcheries are all included in poultry wastes or manure [32].

In this work, attention is paid to enriched biofilm communities that contain bacteria capable of donating electrons to the anode as their final electron acceptor, termed anode-respiring bacteria (ARB). To realize the full potential of MFC technologies, it is important to study the different organisms and mixed communities capable of anodic respiration so that a wider range of metabolic processes can be found, understood, and exploited. This can only be achieved if we expand our search for a new ARB beyond the locations investigated so far. To be more specific, this study assesses the effects of isolated bacteria from anode plates of single-chambered MFC with poultry wastewater samples on electricity generation of the double-chambered MFC.

## 2. Materials and Methods

### 2.1. Collection of Samples and Isolation of Electroactive Bacteria

**Figure 1:**
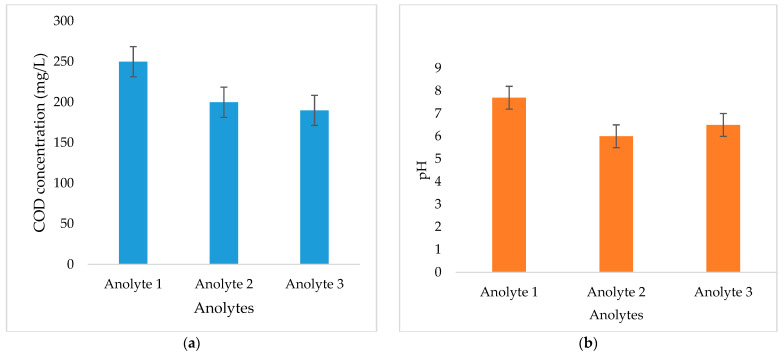
Differences in the used anolytes (**a**) in terms of COD concentration; (**b**) in terms of pH.

Poultry dropping samples were collected near the city of Astana and delivered in sealed plastic bags to the laboratory. Samples were stored in a refrigerator (4 °C) before use. The effluent was prepared by weighing 500 g/L of slurry concentration of poultry manure as feedstock. Pure culture electricigens were isolated from poultry feedstock in single-chambered MFCs and used in double-chamber MFCs for their electroactivity. Replicates were carried out in the study to guarantee the quality of the retrieved results. Three distinct anolytes were used in the investigation, as was previously mentioned (Figure 1). Anolytes were collected at various COD concentrations and related pH levels.

### 2.2. A Single-Chambered MFC Configuration and Operation for the Enrichment of Electroactive Microbes on an Anode

**Figure 2:**
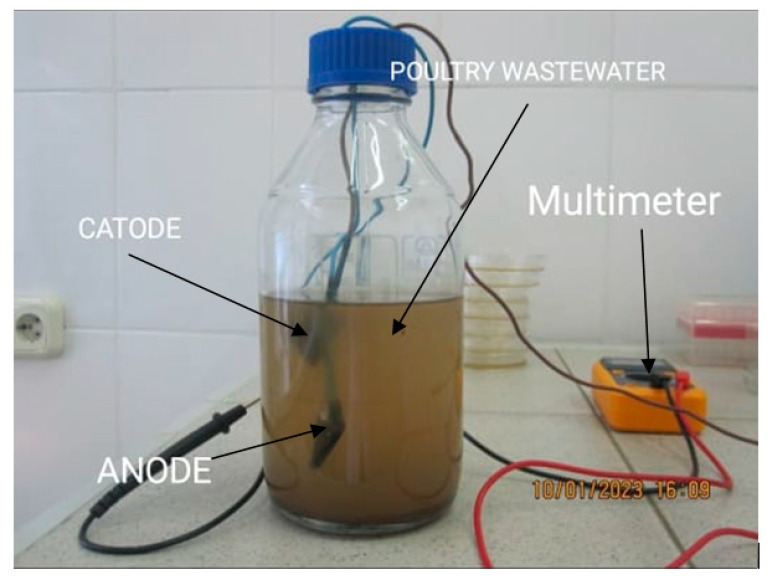
Single-chambered MFC.

The MFC contained 500 mL of poultry wastewater samples and 25 mM acetate as the electron donor for poultry wastewater samples. It is also worth noting that a 1000 mL bottle was used as a container for the media and electrodes (Figure 2). As previously highlighted, graphite electrodes were used in this study as they are considered to be relatively highly conductive, non-reactive, but also inexpensive compared to other electrode materials. The bottle and graphite electrodes were autoclaved before their utilization in the experiments. The current was continuously monitored through the multi-potentiostat and we recorded measurements every day. The temperature was maintained at 32 °C and chambers were mixed constantly with a magnetic stir bar at 150 rpm.

### 2.3. Microbiological Techniques Used for Isolation and Identification of the Electroactive Strain

To isolate microorganisms growing on the surface of the anodes, the electrode surface was washed with a jet of sterile water until visible debris particles were completely removed. Approximately the first millimeter of the graphite electrode (the anode that was in the sludge) was vigorously scraped off with a sterile razor blade in 1.5 mL of phosphate buffer (50 mM) and pH around 7.2, obtaining a suspension consisting of graphite and electrode-associated microbes. The resulting suspension was serially diluted to 10^−6^ and plated on Mueller–Hilton (MH) agar plates. After the incubation period, morphologically distinct colonies were picked up from the Petri plates and restacked in appropriate media, and pure cultures were obtained. Bacterial strains were isolated, grown and maintained on MH agar.

### 2.4. Morphological Characterization

The variety in bacterial form is enormous. The result of adaptive forces that maximize bacterial fitness lead to certain forms. Important biological processes such as nutrition uptake, motility, dispersal, stress resistance, and interactions with other species are influenced by shape. Gram stains were performed according to the procedures described by Merchant and Packer [33], to determine the size, shape, and location of the bacteria.

### 2.5. Genetic Identification of Bacteria

The identification of bacterial isolates was carried out using the method of determining the direct nucleotide sequence of the 16s rRNA gene with the subsequent comparison of nucleotide identity with sequences deposited in the international Genbank database. The PCR reaction was carried out with universal primers 8F (5′-agagttgatgatggctCAG-3′) and 806R (5′-GGACTACGGGGGTATAAT-3′) in a total of 30 μL [34,35]. Identification was performed on a 3730xl DNA Analyzer (Applied Biosystems, Waltham, MA, USA) using a BigDye^®^ Terminator v3.1 Cycle Sequencing Kit (Applied Biosystems, Waltham, MA, USA).

### 2.6. Preparation of Potential Electroactive Strains as Inoculums and Anolyte Substrate for Their Testing in Double-Chamber MFC

The inoculum included a nutrient medium with isolated bacteria. The nutrient medium contained (in g/L): NaHCO_3_ (2.5), NH_4_Cl (0.5), yeast extract (0.2), peptone (0.1), NaH_2_PO_4_ × H_2_O (0.6), KCl (0.1), iron citrate (13.7). sodium acetate (6.8) and lactic acid (1.5) in distilled water, with adjusted pH to 7.2. As an anolyte substrate, synthetic wastewater with glucose, as a carbon source and electron donor, was transferred into a flask that was sparged with CO_2_. Acetate was added as the sole carbon source and electron donor. Acetate (1 g/L) medium, which contained other micronutrients including 1 g/L, NH_4_Cl, 0.28 g/L, KH_2_PO_4_, 0.68 g/L, K_2_HPO_4_, 0.87 g/L, MgSO_4_ × 7H_2_O, 0.1 g/L, CaCl_2_ × 2H_2_O 0.1g/L, NaCl, 0.58 g/L, KCl, 0.74 g/L and vitamin 1 mL/L, was used as anolyte. This medium was injected into an anode chamber by a peristaltic pump (Nanozist tech 5760P) (Gemini Gases, Delhi, India) at different organic loading rates and hydraulic retention times. The multimeter was connected to the MFC using crocodile clips. The average initial COD concentration in the wastewater was 350 mg/L. Synthetic wastewater fed to the bioelectroreactor had a pH ranging from 7.1 to 7.4.

### 2.7. Double-Chamber MFC Configuration and Operation

**Figure 3:**
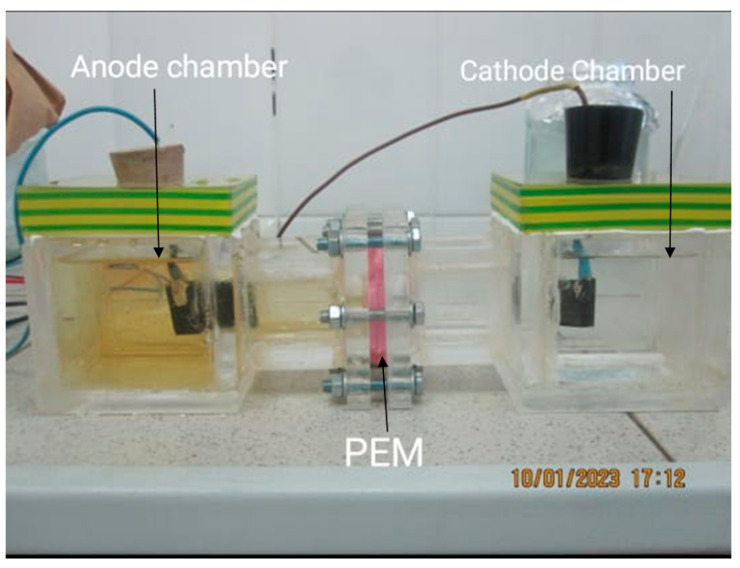
Dual-chambered microbial fuel cell.

Two-chamber MFC constructed by Plexiglas with an internal dimension of 10 × 10 × 5 cm (500 mL) was sterilized by autoclaving at 121 °C for 20 min. A proton exchange membrane was used to separate the anode and cathode chambers (20 cm distance between anode and cathode). To increase the porosity of PEM, it was pretreated. PEM prior to use must be kept in deionized water. Graphite flats (5 cm × 2 cm) without any coating were used as an electrode in the anode and cathode. Both anode and cathode electrodes were positioned in the reactor by copper wires. Copper wires of resistance 100Ὠ were used for connecting circuits. The applied low external resistance (100 ohms) facilitates the electron transfer crossing. Before starting the pilot, the electrodes were pretreated with deionized water for 24 h. The substrate was poured into the anode and cathode compartment, while for the anode part, the inoculum was transferred and this MFC was run for 72 h to investigate the electricity generation. All the steps were repeated for all types of potential electroactive bacteria and abiotics to detect the electricity generation. The double-chambered MFC used in this study is shown in Figure 3.

A total of 40 mL of inoculum was used in the anode compartment and both the anode compartment and the cathode compartment were filled with the substrate to a volume of 400 mL. Both MFC chambers had an arm terminating in a junction with a proton exchange membrane (PEM), such as Nafion, impregnated with a 1% HCl solution for 24 h prior to use in the MFC. Nafion was wrapped between bonds and clamped with forceps. The top opening of each chamber was covered with cotton wool and wrapped with aluminum foil. The electrode materials that were used in this experiment were a graphite plate, and this electrode was cut into pieces of 5 cm × 2 cm. This was turned on for 72 h to show power generation. The anode cell was sparged with sterile high-purity N_2_ for 15 min to remove oxygen before analysis. All steps were repeated for all types of electroactive bacteria and abiotic materials to detect electricity generation. Using a multimeter, the power generation voltage readings from the MFC were recorded. Voltage measurements were recorded in millivolts (mV) which were taken over 72 h.

### 2.8. Calculation of Current and Power Density

The current was calculated from the fixed external resistance (R = 100 Ὠ) and cell voltage, according to Ohm’s law. Cell voltage (mV) was continuously measured using a digital multimeter (UK-831LN). The multimeter was connected to the MFC using crocodile clips. The measurement of voltage was recorded in millivolts (mV) and the reading was taken for 24 h. Power (mW/m^2^) was calculated by using the formula:

(1) $$P=\frac{V^{2}}{R\times A}$$

where V (mV) is the output voltage, A (cm^2^) is the surface area of the anode, and R (Ὠ) is the external resistance. The current obtained was normalized to the surface area (20 cm^2^) of the anodic electrode.

### 2.9. Estimation of COD Removal and Coulombic Efficiency

COD removal was used to assess the efficacy of the double-chamber MFC. COD was assessed in the samples using a spectrophotometer (Hach LANGE DR 3900, Hach, Berlin, Germany) set at 600 nm [36]. COD removal efficiency [%], was calculated based on the initial and final COD. The Coulombic efficiency (CE) enables estimation of the efficacy of electron donor oxidation in producing current. The following equation (Equation (2)) was used to compute Coulombic efficiency (CE), which is the fractional recovery of electrons from the substrate:

(2) $$\mathrm{CE}=\frac{M\;\times \;I}{\mathrm{nvFv}\Delta \mathrm{COD}}\times 100$$

where M is the molecular weight of the substrate, I is the average current (mA), F is Faraday’s constant (96,485 C mol^−1^), n is the number of electrons exchanged per mole of oxygen (4 mol e^−^ mol^−1^), v is the MFC volume (L), and ∆COD is the change in COD over time t (g L^−1^).

## 3. Results

### 3.1. Voltage and Current Generation in Single-Chambered MFC

**Figure 4:**
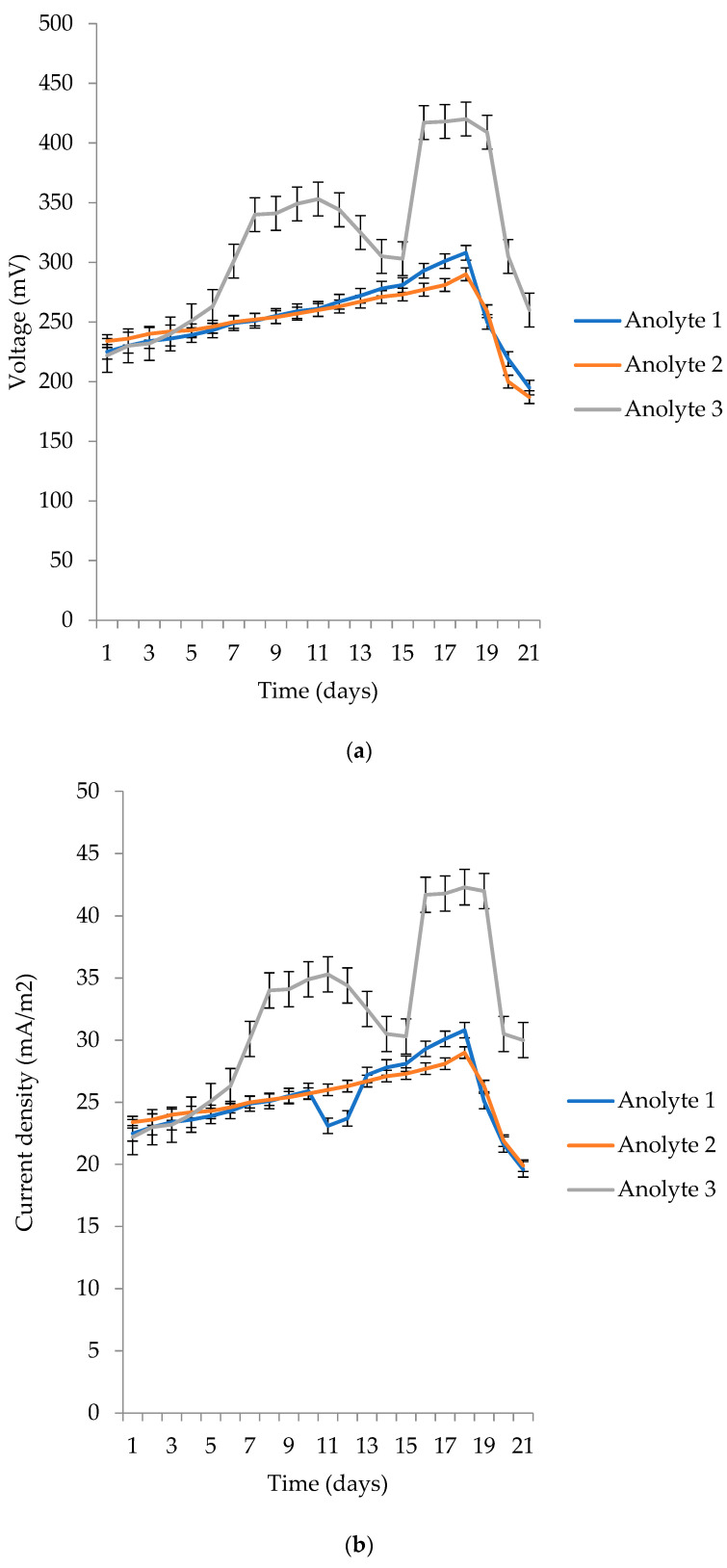
Voltage (**a**) and current (**b**) generation in a single MFC of poultry wastewater in the single-chamber MFC for 21 days.

In the single-chambered MFC, wastewater from a chicken farm was used as an anolyte in the search for electroactive microorganisms. The goal of the study was to quantify the energy produced by the single-chamber MFC’s low power output, which did not require any additional energy to operate. Figure 4 displays the outcomes of the MFC’s activity throughout the course of 21 days, specifically the multimeter’s measurements of current and electric potential (V). Fuel cells can be operated at the best current or voltage ranges to maximize power density (PD) generation, according to the power–current properties of MFC. At the start-up stage of a single-chamber MFC with three poultry wastewater anolytes, the performance reached a maximum voltage of 420 mV and a maximum current density of 41.8 mA/m^2^ in the period of 17–19 days (Figure 4). Furthermore, the voltage during the test of all three analytes began to gradually decrease, which indicated the depletion of nutrients for microorganisms present in the poultry wastewater. After the optimal anolyte, which showed the highest voltage, was determined, possible electroactive cultures were isolated by inoculating a smear taken from the surface of a graphite anode onto Petri dishes with MH medium.

**Figure 5:**
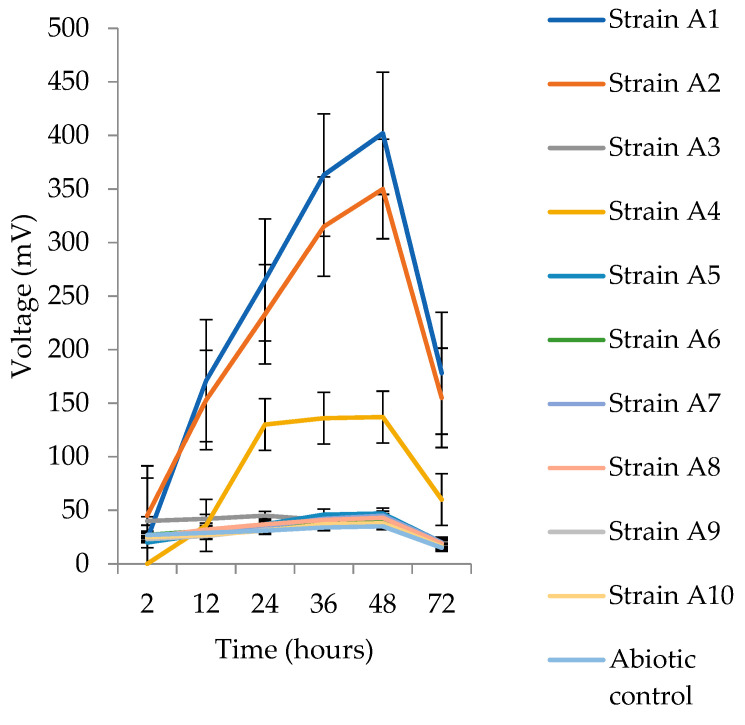
Testing pure bacterial cultures isolated from single-chambered MFC with poultry wastewater in double-chambered MFC for electricity generation for 72 h.

Figure 5 shows the results of the electrical generation by isolated bacterial cultures in the double-chamber MFC. Ten isolates (A1 A2, A3, A4, A5, A6, A7, A8, A9, and A10 bacterial cultures) were isolated and tested for electrical activity over 72 h. Three separate tests were run on each isolate to determine their ability to produce electricity.

**Figure 6:**
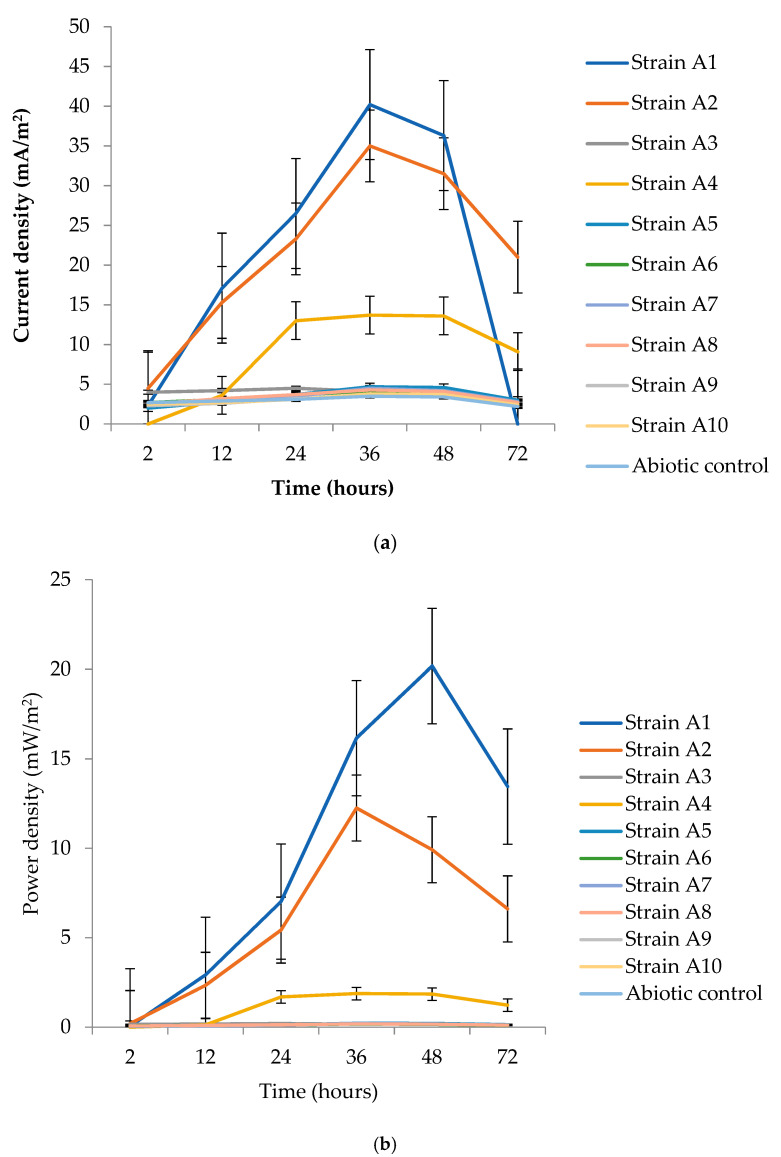
Current density (**a**) and power density (**b**) of isolated strains and abiotic control in a double-chambered MFC over 72 h.

Based on the investigations from the isolated bacterial cultures, strains A1 and A2 produced the highest voltage readings of 402 mV and 350 mV, respectively, and significantly differed from the strains of the other eight bacterial isolates and controls. The lowest value of 35.03 ± 1.15 mV obtained for electricity generation among the ten types of bacterial strains was for strain A6. Furthermore, in parallel, an analysis was performed on the current density and power density for a complete characterization of electrical generation. Figure 6 shows indicators of current density and power density in the double-chamber MFC.

The MFC with strain A1 performed much better than the MFC with a sterile medium, with a maximum power density of 16.16 ± 1.02 mW/m^2^. Strain A2 displayed high current and power densities of 35 ± 1.12 mA/m^2^ and 12.25 ± 1.05 mW/m^2^, respectively.

### 3.2. COD Removal and Coulombic Efficiency

**Figure 7:**
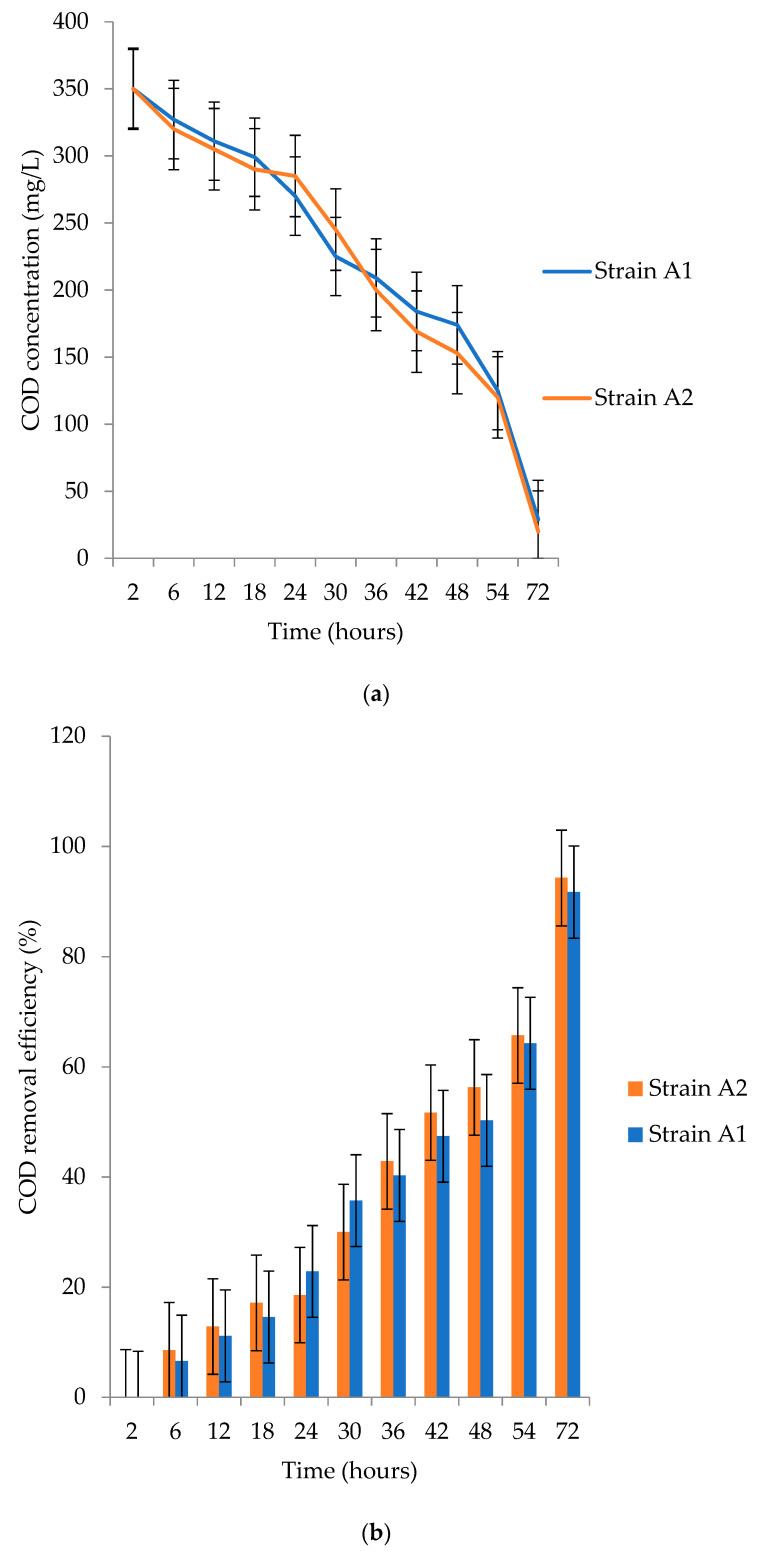
COD analysis results. (**a**) COD concentration (mg/L) and (**b**) COD removal efficiency within 72 h achieved from strains A1 and A2.

COD removal and Coulombic efficiency were observed in the two representative strains. To detect the removal efficiency of COD, samples were taken from the effluent anode chamber. The average initial COD concentration in wastewater was 350 mg/L (Figure 7).

According to the COD removal efficiency results after 72 h, strains A1 and A2 degraded 94.28% and 91.71%, respectively, of the organic substrate. Over 72 h, strains A1 and A2 had electron donor oxidation efficiencies of 54.1% and 60.67%, respectively. With the reduction of COD, the CE increased, indicating microbial electroactivity. The CE values of 10% and 3.5% were obtained in the MFC with representative strains A1 and A2, respectively.

### 3.3. Phenotypic Characterization of Selected Strains

**Figure 8:**
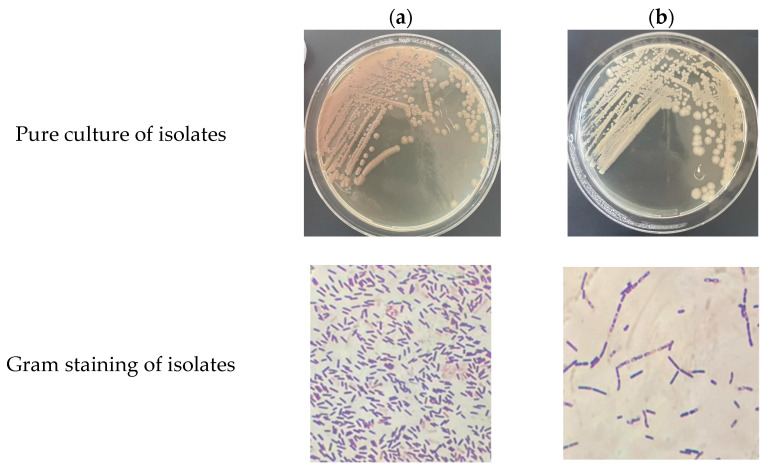
Identical pure cultures of the most efficient isolates on plates with nutrient agar medium and their microscopic characteristics: (**a**) A1 isolate and (**b**) A2 isolate.

Then, the obtained pure cultures of bacteria were cultured on Mueller–Hilton agar and their phenotypic characterization was carried out. Colonies of strain A1 were observed to be opaque, dark yellow, smooth, and shiny when grown in a nutrient medium (Figure 8). Strain A1 bacteria are Gram-positive long rods in their structure. Colonies of strain A2 have a fuzzy white or light-yellow tint, and are opaque, spherical, and rough, with jagged edges. Bacteria of strain A2 are Gram-positive bacilli with blunt ends and oval terminal spores.

### 3.4. Genetic Identification of Selected Strains

All 10 isolates were genetically identified: A1—*Lysinibacillus sphaericus*, A2—*Bacillus cereus*, A3—*Pediococcuspentosaceus*, A4—*Lactococcus lactis*, A5—*Lactobacillus casei*, A6—*Lactobacillus rhamnosus*, A7—*Arthrobacter histidinolonovorans*, A8—*Enterobacter cloacae*, A9—*Rhodococcuserythropolic*, A10—*Pseudomonas fluorescens.* Strains A1—*Lysinibacillussphaericus* and A2—*Bacillus cereus*, turned out to be electroactive bacteria. Subsequently, using the sequences of the 16S rRNA gene, these two types of cultivated microbes were identified, and showed the highest electrogenic activity. Bacterial culture A1 was identified as *Lysinibacillus sphaericus* and bacterial culture A2 as *Bacillus cereus*. Table 1 provides a summary of the results from the identification of nucleotide sequences in the international database BLAST algorithm.

The energy performance results of the isolates were compared with known industrial electroactive bacterial strains tested in double-chambered MFCs (Table 2).

## 4. Discussion

The environmental load of wastewater and other waste streams has increased due to an increase in untreated environmental outputs from industries and an increase in the human population. The activated sludge process, which requires aeration and is consequently energetically and financially expensive, is one of the most widely used wastewater treatment techniques [42]. The recovery of waste materials as resources is made easier by the present movement toward a circular economy. The complexity of wastewater is also growing daily, in addition to its volume. As a result, technologies for wastewater treatment that are both affordable and sustainable must be developed. Moreover, electrochemical technologies, such as fuel cells, exhibit tremendous future promise as power technologies for alternative energy sources due to the depletion of natural resources. The MFC, which generates bio-electricity from various organic fuel sources, is one such promising invention. In order to create power through waste treatment, MFCs use electroactive bacteria to extract chemical energy from used organic molecules [43].

This study worked on the electrochemical characterization of bacteria isolated from poultry wastewater. Possible electroactive cultures were isolated by inoculating a smear collected from the surface of a graphite anode onto Petri dishes with MH medium after the best anolyte, which displayed the highest voltage, was identified. As a consequence, 10 isolates of the bacterial cultures (A1 A2, A3, A4, A5, A6, A7, A8, and A10) were discovered. These isolates underwent a 72 h test for electrical activity. Of the 10 isolated pure cultures of microorganisms, 7 were facultative anaerobes. The remaining three species of bacteria of the genus Arthrobacter, Rhodococcus, Pseudomonas were obligate aerobes. Among the seven facultative anaerobic bacteria, bacteria of the genera Lysinibacillus and Bacillus cereus, which are the closest in the phylogenetic tree and have similar metabolic processes, showed electroactivity. Both genera of bacteria are capable of high consumption of acetate. *Lysinibacillus sphaericus* comprises a group of motile Gram-positive spore-forming bacilli. Members of this group are characterized by their terminal endospore, the capability to utilize acetate as the sole carbon source, and the presence of lysine and aspartic acid in their cell wall peptidoglycan. According to literature sources, bacteria *Lysinibacillus sphaericus* are capable of adsorbing toxic metals (cadmium, lead, arsenic, mercury, chromium) and precious metals. The metal ions become attached to the functional groups or the protein layer, followed by the binding of metal ions to the reactive groups present on the bacterial cell wall; the internalization of metal ions occurs inside the cell. Transcriptomic, proteomic, and electrochemical analyses show that the electrode respiration of *Lysinibacillussphaericus* mainly depends on electron mediators, and c-type cytochromes may be involved in its respiration. Extracellular electron transport (EET) is a key driving force in biogeochemical element cycles and microbial chemical–electrical–optical energy conversion on Earth. Gram-positive bacteria are ubiquitous and even dominant in EET-enriched environments. However, attention and knowledge of their EET pathways are largely lacking. The Gram-positive bacterium Lysinibacillus has extremely long cells (>1 mm) and conductive nanowires, promising a unique and enormous role in the microenvironments where it lives. Furthermore, the isolation of the potential electrogene makes it possible to develop an electrochemical strategy for connecting and forming the surrounding microbial community on a minimal scale. Spore-forming bacteria belonging to the genus Bacillus have the ability to release flavins. These flavins allow Bacillus sp. to mediate electron transfer to electrodes in MFCs and provide increased power generation in microbial consortia with Gram-negative bacteria or yeast. The incorporation of Bacillus cells into anaerobic sludge has also had a significant impact on power generation in MFCs. By promoting the formation of an electroactive biofilm and inhibiting methanogenesis, Bacillus cereus enhances current production in the MFC.

As noted from the results, in the first 17 to 19 days of operation, the performance of a single-chambered MFC using three poultry wastewater anolytes reached a maximum voltage of 420 mV and a maximum current density of 41.8 mA/m^2^. As said before, an MFC may produce power directly from a wide range of organic or inorganic chemicals by acting as a catalyst and using microbes. Traditionally, fuel cells use an oxidant at the cathode and a fuel at the anode to transform chemical energy into electrical energy. Electricity is generated as a result of the liberated electrons and protons moving through an external circuit. MFCs use an organic matter and microbial fuel solution, with the anode and cathode separated by an ion exchange membrane [44]. However, the direct passage of electrons from the bacterium to the anode significantly reduces efficiency. Therefore, exogenous mediators such as thionine, methyl viologen, and humic acid are utilized in electrochemically inactive microbial cells. These serve as electron shuttles, diffusing electrons to the anode, allowing them to discharge, and then diffusing them back to the bacterial cells. However, these mediators are extremely expensive and harmful to microorganisms [45].

In addition, tests of two isolated bacterial cultures designated strains A1 and A2, produced the greatest voltage outputs (402 mV and 350 mV, respectively) and were significantly different from the strains of the other eight bacterial isolates and controls. Ten different bacterial strains were tested, and strain A6 produced the least electricity at 35.03 mV. In addition, a parallel investigation of the current and power densities was carried out in order to characterize electrical generation completely. When compared to MFCs using a sterile medium, strain A1 dramatically enhanced performance, with a maximum power density of 16.16 1.02 mW/m^2^. In terms of current density and power density, strain A2 displayed high values of 35 ± 1.12 mA/m^2^ and 12.25 ± 1.05 mW/m^2^, respectively. Koffi and Okabe [46] claimed that an MFC may commonly provide power densities between 1 and 2000 mW m-^2^. So far, the low voltage or power issue has been solved by simply connecting numerous MFCs in series or parallel. Although a unit of serially stacked MFCs may deliver a larger voltage, it has frequently been found to be challenging and unsuccessful because individual the tendency of MFC units to switch polarity due to fuel shortages causes a sizable overall voltage decay. A stacked polarized capacitor has recently been charged using individual MFC units connected to an MPPT system in an effort to manage and reduce voltage reversal occurrence. However, only voltages between 2 and 3 V could be increased using this technical method [46].

According to the results of COD removal efficiency after 72 h, strains A1 and A2 converted 91.71% and 94.28% of the organic substrate, respectively. The electron donor oxidation efficiencies of strains A1 and A2 for 72 h were 54.1% and 60.67%, respectively. Additionally, the results showed that Coulombic efficiency increased when COD was decreased, indicating an increase in microbial electroactivity. The representative strains A1 and A2 were then selected for further investigation. It should be underlined that COD is a measurement of the amount of oxygen used during the oxidation of oxidizable organic matter when a potent oxidizing agent is present. The number of organic compounds in wastewater are typically estimated indirectly using this method. High COD indicates the presence of all organic matter types, both biodegradable and nonbiodegradable, and subsequently the level of pollution in the water. Because of this, COD can be used to detect organic pollution in surface waters [47]. The results of COD removal were found to be comparable to those of Li et al. [48], whose work sought to identify and characterize a COD-degrading bacterium that can efficiently break down slaughter wastewater. In their investigation, six COD-degrading bacteria were found in the sludge and wastewater from the Hunan meat industry’s slaughterhouses. *Bacillus velezensis* was found and categorized as the strain with the highest COD degradation rate through morphological observation and 16S rDNA sequence analysis, reaching 11.80%. It should be mentioned that the potassium permanganate method was used to assess the COD breakdown rate of each strain.

The acquired pure bacterial cultures were characterized phenotypically after being inoculated on Mueller–Hilton agar. A variety of non-picky organisms can be grown on the Mueller–Hinton agar, which is a non-selective, non-differential medium. It is referred to as a “loose” agar, which works better than other forms of media to mediate the rate of antibiotic diffusion. On a nutritional medium, isolate A1 colonies are opaque, dark yellow, smooth, and shiny. The bacteria from the A2 isolate are rod-shaped and Gram-positive. Bacteria that are both sporogenous and non-sporogenous make up the diverse group known as Gram-positive rods. The genus *Bacillus* is made up of aerobic, sporogenous organisms that are Gram-positive. There are several species in this, but Bacillus anthracis, the agent of anthrax, is the most significant from a medical and veterinary standpoint. Isolate A2 colonies are rounded and rough, have wavy edges, are opaque, and are a fuzzy white or slightly yellow tint. Rod-shaped and Gram-positive bacteria make up the A2 isolate. It is also crucial to emphasize that the production of alternative fuels to replace conventional fossil fuels has become necessary due to the rapidly diminishing concentration of fossil fuels and the increasing global demand for energy [49]. This is carried out in order to counter the increased deposition of greenhouse gases in the atmosphere, which has resulted in significant climatic changes. Rising temperatures and sea levels are just two of the potentially disastrous effects of these changes.

Additionally, the findings of the double-chambered MFC tests on the isolates’ energy performance were contrasted with those of well-known electroactive bacterial strains (Table 2). Based on the results, it is clear that the density of the *Lysinibacillus sphaericus* A1 and *Bacillus cereus* A2 strains were several times less than that of the *Shewanella oneidensis* MR-1 production strain and less than the well-known *Lysinibacillus spharicus* VA5, *Lysinibacillus sphaericus* D-8. However, the Coulombic efficiency was higher than that of the *Lysinibacillus sphaericus* VA5, which was explained by the effectiveness of the removal of the COD from the *Lysinibacillus sphaericus* A1 strain. It should be noted that the electrochemical and Coulombic efficiency of the selected *Lysinibacillus sphaericus* A1 strain was higher than that of the well-known *Corynebacterium* sp. MFC03 strain. Furthermore, the *Bacillus cereus* A2 strain showed severe electrical activeness and was not inferior in producing current density and power compared to such an electric active strain as *Corynebacterium* sp. strain MFC03. These comparative indicators of the above strains indicate a high electrical potential of the *Lysinibacillus sphaericus* A1 and *Bacillus cereus* A2.

## 5. Conclusions

In this study, a single-chamber MFC containing poultry wastewater was used to isolate putative electroactive microorganisms. Two Gram-positive bacterial strains, *Lysinibacillus sphaericus* (A1) and *Bacillus cereus* (A2), were isolated and their electrogenic capacities were investigated. To be more precise, this study used a double-chambered MFC to enrich graphite anodes with different electroactive bacteria. The isolated strain A1 *(Lysinibacillus sphaericus)* was electrochemically characterized in the study as a facultative anaerobic electrogenic bacterium. The COD removal efficiency over 72 h, according to the *Lysinibacillus sphaericus* A1 findings, was 94.28%. A maximum power density of 16.16 mW/m^2^ and a CE of 10% were obtained in the MFC. *Bacillus cereus* A2, the second novel electroactive isolate, likewise produced promising outcomes. *Bacillus cereus* had a 72 h COD removal efficacy of 91.71%, and a maximum power density of 12.25 ± 1.05 mW/m^2^. As a result, the results of this study point to *Lysinibacillus sphaericus* A1 as a strong candidate for the design and development of MFCs for energy production. These isolated electroactive bacteria were placed in long-term storage for further, more detailed studies. To improve the efficiency of isolated electrogens, other carbon sources included in artificial or real wastewater can be evaluated. Finding more effective electrogenic organisms requires further research into the diversity of accessible electrogenic microbes as well as the methods employed to transfer extracellular charge to electrodes. This study will contribute to a better understanding of the electrogenic potential of pathogens present in the avian microbiome, which is currently largely uncharacterized. The findings of this study also broaden the knowledge of exoelectrogens for energy generation, and the vast range of substrates used by the strains raises the possibility for MFC applications in waste management and renewable energy production.

## Figures and Tables

**Table 1:** The results of identification by analysis of the nucleotide sequence of the 16S rRNA gene.

Strains	GenBank Inventory Number (Accession Number)	Name of the Strain	% Match
Strain A1	NR_112627.1	*Lysinibacillus sphaericus*	100%
Strain A2	NR_115526.1	*Bacillus cereus*	100%

**Table 2:** Summary of the comparative analysis with known industrial electroactive bacterial strains tested in double-chambered MFCs.

Bacterial Strain	Voltage Generation	Current Generation	Power Generation	COD Removal Efficiency	Coulombic Efficiency	Substrate	External Resistance	Operational Days	Reference
*Lysinibacillus sphaericus* A1 Accession number NR_112627.1	0.4 mV	40.2 mA/m^2^	16.16 mW/m^2^	94.28%	10%	acetate	100 Ω	3	this work
*Lysinibacillus sphaericus* VA5 Accession number HE648059	0.7 V	270 mA/m^2^	85 mW/m^2^	70%	between 1 and 3%	glucose	100 Ω	12.5	[37]
*Lysinibacillus sphaericus* D-8 Accession number KC691284	0.4 V	142 mA/m^2^	92 mW/m^2^		12.69%	lactate	1000 Ω	3	[38]
*Bacillus cereus* A2 Accession number NR_115526.1	0.3 mV	35 mA/m^2^	12.25 mW/m^2^,	91.71%	3.5%	acetate	100 Ω	3	this work
Bacillus cereus DIF1 Accession number MH351294.1	0.3 V	37.05 mA	-	-	-	-	1000 Ω	1.25	[39]
*Shewanella oneidensis* MR-1 Accession number NC_004347.2	0.7 mV	2700 mA/m^2^	578 mBt/m^2^	83%	-	acetate	1000 Ω	8	[40]
*Corynebacterium* sp. strain MFC03	-	33.6 mA/m^2^	7.3 mW/m^2^	80.1%	5.9%	glucose with 0.1 mM anthroquinone-2,6-disulfonate (AQDS)	1000 Ω	3	[41]

## Data Availability

The data that support the findings of this study are available from the corresponding authors upon reasonable request.
